# Compensatory density feedback of *Oncomelania hupensis *populations in two different environmental settings in China

**DOI:** 10.1186/1756-3305-4-133

**Published:** 2011-07-13

**Authors:** Guo-Jing Yang, Xiao-Nong Zhou, Le-Ping Sun, Feng Wu, Bo Zhong, Dong-Chuan Qiu, Jürg Utzinger, Corey JA Bradshaw

**Affiliations:** 1 Jiangsu Institute of Parasitic Diseases, Meiyuan Yangxiang 117, Wuxi 214064, People's Republic of China; 2 National Institute of Parasitic Diseases, Chinese Center for Disease Control and Prevention, Shanghai 200025, People's Republic of China; 3 WHO Collaborating Center for Malaria, Schistosomiasis and Filariasis; Key Laboratory of Parasite and Vector Biology, MOH, People's Republic of China; 4 Institute of Parasitic Diseases, Sichuan Center for Disease Control and Prevention, Chengdu 610041, People's Republic of China; 5 Department of Epidemiology and Public Health, Swiss Tropical and Public Health Institute, CH-4002 Basel, Switzerland; 6 University of Basel, CH-4003 Basel, Switzerland; 7 The Environment Institute and School of Earth and Environmental Sciences, University of Adelaide, South Australia 5005, Australia; 8 South Australian Research and Development Institute, Henley Beach, South Australia 5022, Australia

## Abstract

**Background:**

The most recent strategy for schistosomiasis control in the People's Republic of China aims to reduce the likelihood of environmental contamination of schistosome eggs. Despite considerable progress, it is believed that achievements would be further consolidated with additional intermediate host snail control measures. We provide an empirical framework for discerning the relative contribution of intrinsic effects (density feedback) from other extrinsic drivers of snail population dynamics.

**Methods:**

We set up experiments in two study locations to collect reproduction data of *Oncomelania hupensis*, the intermediate host snail of *Schistosoma japonicum*. We applied a set of four population dynamic models that have been widely used to study phenomenological time-series data to examine the properties of demographic density feedback patterns from abundance data. We also contrasted the obtained results with the component feedback of density on survival rate to determine whether adult survival was the principal driver of the demographic feedback observed.

**Results:**

Demographic density feedback models (Ricker- and Gompertz-logistic) accounted for > 99% of Akaike's information criterion model weight, with the Gompertz ranking highest in all *O. hupensis *population groups. We found some evidence for stronger compensatory feedback in the *O. hupensis *population from Sichuan compared to a Jiangsu population. Survival rates revealed strong component feedback, but the log-linear relationships (i.e. Gompertz) had less support in the demographic feedback analysis.

**Conclusions:**

Our findings indicate that integrated schistosomiasis control measures must continue to reduce parasite abundance further because intermediate host snail populations tend to grow exponentially at low densities, especially *O. hupensis *populations in mountainous regions. We conclude that density feedback in adult survival is the principal component contribution to the demographic phenomenon observed in the population fitness (*r*)-abundance relationship.

## Background

In the People's Republic of China (P.R. China), schistosomiasis caused by the blood fluke *Schistosoma japonicum *has a documented history of more than 2,000 years [1,2]. The first large-scale surveys done in the mid-1950s suggested that the disease was endemic in 12 provinces located along and south of the Yangtze River. More than 10 million people were infected, causing considerable morbidity and even mortality [3,4]. Hence, a national schistosomiasis control programme was launched, placing particular emphasis on the control of the intermediate host snail *Oncomelania hupensis*, including environmental management and chemical mollusciciding [2-6]. As a result, snail-infested areas have been reduced from approximately 14,320 km^2 ^in the mid-1950s to 3,720 km^2 ^in 2008 [6,7].

Recently, a comprehensive strategy was proposed with the ultimate aim to reduce further the likelihood of contamination of the environment with schistosome eggs. This integrated control strategy consists of health education, access to clean water and adequate sanitation, mechanization of agriculture and fencing of domesticated bovines, along with preventive chemotherapy [8,9]. The main rationale for implementing this new strategy is that schistosomiasis is an environmentally mediated disease, and that it is difficult to eliminate all snail habitats, particularly in lake and marshland regions [10]. However, this new strategy alone does not succeed in eliminating or substantially reducing the incidence of schistosomiasis [11], especially in mountainous regions where suitable snail habitats persist. Additional control measures are needed, such as mollusciciding, which is a time-consuming and costly strategy, because large fluctuations in snail abundance [12,13] can arise from flooding [14,15]. Hence, measures for increasing the effectiveness of mollusciciding, which in turn reduce intermediate host snail abundance and limit the likelihood of re-emergence of schistosomiasis, are required [16].

For the effective control of *O. hupensis *populations, a fundamental step is a deeper understanding of the snail's intrinsic population dynamics because these properties influence the rate of recovery after withdrawal of snail control [1]. There is, however, a paucity of information describing even basic population dynamics for this species, which severely limits our understanding of the processes of schistosomiasis transmission, and hence hampers the development of effective control approaches. here is a general consensus among ecologists that account must be taken of intrinsic and extrinsic population controls [17] when analysing time-series data, and growing emphasis is placed on determining the degree of interaction between the two aspects driving fluctuations in abundance [18-22]. Intrinsic control normally operates via density feedback whereby *component *vital rates (e.g. survival and fertility) and/or individual fitness change in response to population density [23]. However, vital rates can respond differently to density changes, meaning that the influence of density on the rate of population growth (termed *demographic *feedback) as measured by changes in abundance should reflect the net contributions of all component vital rates and extrinsic perturbations [24]. Extrinsic processes include stochastic environmental pressures and human control activities that affect population density, but are not directly affected by population density themselves [22].

Previous research pertaining to *O. hupensis *focused mainly on the environmental conditions correlated with snail abundance, and hence particular emphasis was placed on elucidating extrinsic influences. Similar to mosquito vector species [25-27], snail population dynamics can also exhibit strong density feedback, and the form and relative strength of feedback might differ markedly among populations given the strong genetic differentiation observed in intermediate host snails [28,29]. Failure to take intrinsic dynamics into account can lead to an over-estimation of the medium- to long-term effectiveness of density control methods, such as mollusciciding or habitat modification [23].

Here we provide an empirical framework for discerning the relative contributions of intrinsic drivers of snail population dynamics for a better understanding of the eco-epidemiology and control of schistosomiasis. We first examine the evidence for, strength and form of phenomenological (demographic) density feedback operating in focal snail populations based on a series of biological experiments under quasi-field conditions. We also test whether the intrinsic dynamics of *O. hupensis *follow a Gompertz-like compensatory feedback, which is consistent with organisms having high turn-over rates such as insects [30,31]. The Gompertz model and its analogues express population growth (*r*) or vital rates such as survival (*s*) as a negative log-linear relationship with density, with high *r *or *s *at low densities which decline rapidly as population size increases and then tapers to an asymptote [23]. Examining the patterns of component feedback in snail survival provides insight into the principal drivers of the strength and form of the demographic response. The relevant elements derived from the intrinsic model will provide an ecologically based evidence to formulate cost-effective control strategies towards schistosomiasis elimination, and to ensure the lowest transmission risk or density of *O. hupensis *[23].

## Methods

### Study site

**Figure 1:**
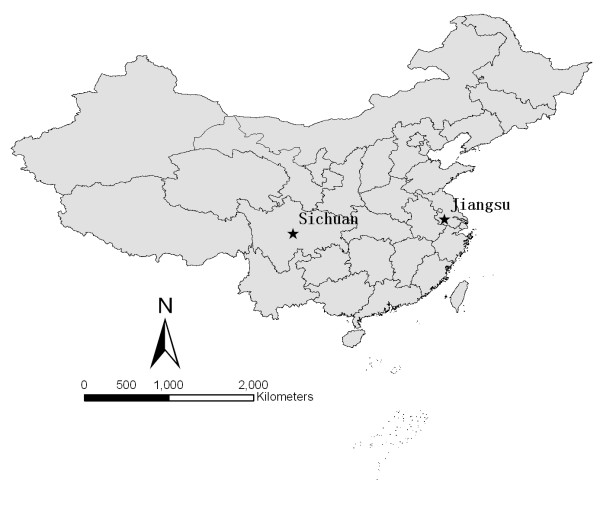
**Study sites in Jiangsu and Sichuan provinces, People's Republic of China**.

To deepen our understanding of intrinsic effects on *O. hupensis *abundance patterns, snail reproduction experiments were done in two different settings: a marshland at Zhang Jiagang, Jiangsu province (119° 30' E longitude and 31° 50' N latitude) and a mountainous region in Pujiang county, Sichuan province (103° 24' E, 30° 18' N) (Figure 1).

### Snail experiments

**Figure 2:**
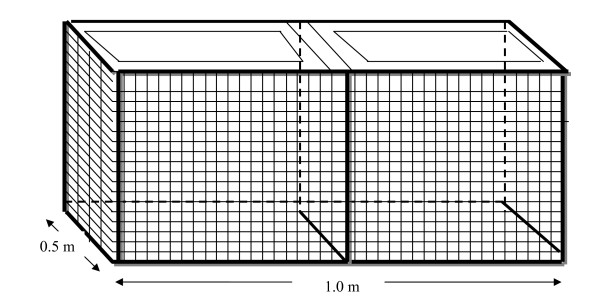
**Schematic diagram of a snail-raising cage**. The frame of the cage is made of crude wire mesh. Inner components of the frame are covered by nylon yarn silk with a mesh size of 40 μm, thus preventing the leaking of snail eggs.

In September 2008, we collected *O. hupensis *from each study site, noting the sex ratio (male:female) from a random sample of 500 snails which was close to 1:1 at both sites. In late 2008, we marked randomly selected adult snails from each study site (on the shell, using waterproof marker pens), and placed them into individual 0.5-m^2 ^cages with soil covering the bottom (Figure 2). According to long-term experience with snail surveillance as part of the national schistosomiasis control programme, densities fluctuate between 10 and 800 snails per m^2 ^[32,33]. We therefore adjusted experimental snail densities from 2 to 1,000 (i.e. 2, 4, 6, 8, 10, 20, 40, 80, 100, 150, 200, 400, 800 and 1000) per 0.5-m^2^. In those cages where 40 snails or less were kept, we placed equal numbers of male and female snails into individual cages to avoid potential sex biases. We kept snail cages at approximately 'natural' soil moisture conditions throughout the year to mimic normal variation. We kept the first generation of snails in cages until late June because the period of peak egg production of *O. hupensis *is around March and April, and generally lasts until June. In June 2009, we counted the snails and removed the marked adults (both dead and alive). We estimated the second generation of snails by counting them in November 2009.

In late September 2009, we repeated the experiment until the following year. We randomly selected adult snails in the second experiment from the new generation of snails obtained in the first experiment. We assembled the data obtained from the two experiments and subjected them to a pooled analysis.

### Analysis

#### Demographic density feedback

Although there are many potential mathematical simplifications of complex population dynamics in time-series analysis (e.g. see references [34,35]), we used an *a priori *model-building strategy to arrive at a set of four population dynamics models commonly used to describe phenomenological time-series data [23,30]. Density-independent models assume constant growth without the influence of density. We applied two density-independent models: (*i*) random walk with a long-term growth rate (intercept) *r_m _*= 0 (RW; equation 1) and (*ii*) exponential growth with a constant (non-zero) value of *r_m _*(EX; equation 2). In contrast, density-feedback models consider *r *as a linear function of *N_t_*; we used a stochastic form of the Ricker-logistic model (RL; equation 3), and a stochastic Gompertz-logistic model with log-transformed *N_t _*and long-term, average carrying capacity *K *(GL; equation 4).(1)(2)(3)(4)

where *N_t _*= snail population size at time *t, r *= realized population rate of change, *r_m _*= maximum rate of population change, *K *= carrying capacity and process error *ε_t_*~ *Normal*(0, *σ*^2^). We fitted all models on the basis of maximum-likelihood using linear regression.

#### Component density feedback

We applied analogues to the EX, RL and GL models for snail survival rate (*s*) analysis. We did not test a zero-intercept model analogous to the demographic feedback RW model because there is not an *a priori *expectation of a particular constant, density-independent survival rate [30]. These contrast the three competing hypotheses of density-independent survival (equation 5), survival declining linearly with density (equation 6), and survival declining log-linearly with density (equation 7), respectively. We used the logit transformation to normalise the *s *response prior to fitting the three linear models:(5)(6)(7)

where *n_l _*= number of live snails, *n_d _*= number of dead snails, *n_i _*= initial number of snails (*n_l _*+ *n_d_*), *s_m _*= the mean or maximum survival rate (intercept), *β*= coefficient (slope) of the abundance (*n_i_*) parameter and *ε *= process error (as described above).

#### Model comparison

To rank all models, we calculated Akaike's information criterion corrected for small sample sizes (AIC*_c_*) [36,37], the difference between the model's AIC*_c _*and that of the top-ranked model *i *(Δ*_i_*, ΔAIC_c_; equation 8), and the relative model weights (*w_i_*; equation 9) [37]. Thus, the strength of evidence (*w*AIC*_c_*) for any particular model varied from 0 (no support) to 1 (complete support) relative to the entire model set of *M *candidate models. We did all analyses using the open-source R statistical program, version 2.12.1 [38].(8)(9)

We also estimated each model's goodness of fit using the percentage of deviance explained (%DE) relative to the null (intercept-only) model.

## Results

### Breeding patterns of *O. hupensis*

**Figure 3:**
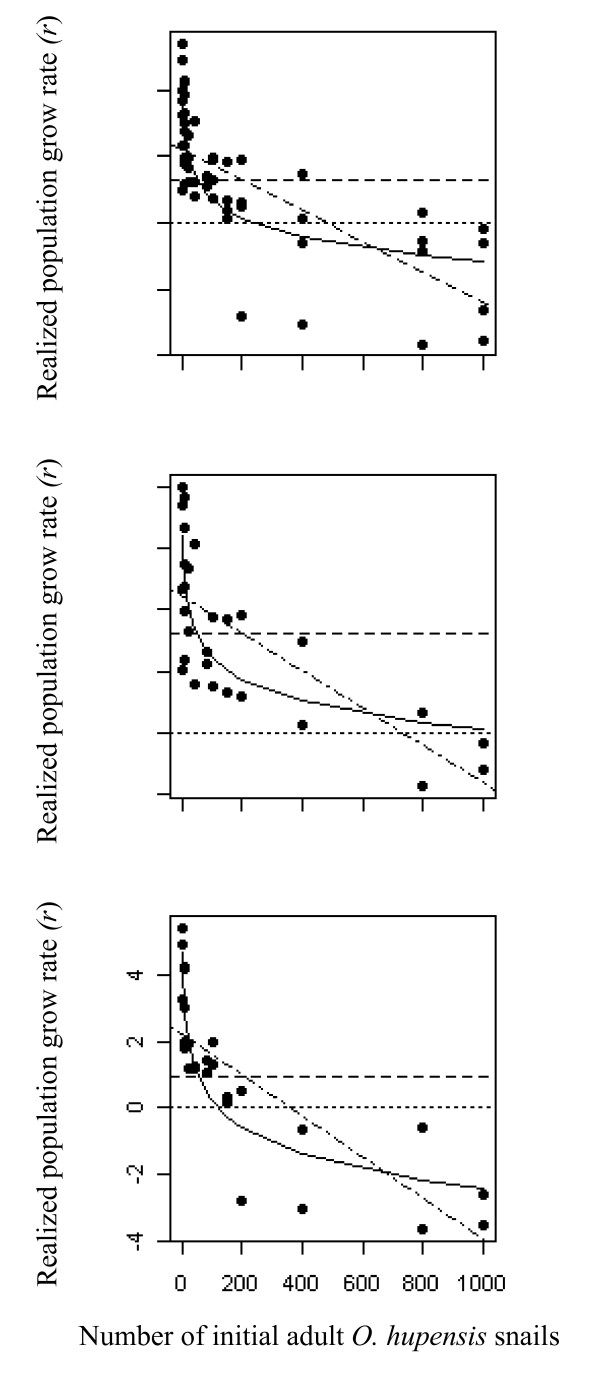
**Four population dynamics models examining the relationship between population growth rate (*r*) and density for the pooled data (upper), Jiangsu (middle) and Sichuan (lower) populations**. See also Table 1. Dotted line, random walk (RW); dashed line, exponential model (EX); dot-dash line, Ricker-logistic (RL); solid line, Gompertz-logistic (GL). See text for details.

We collected the first and second generations of *O. hupensis *snails over the two study years (2009 and 2010) in both sites (Jiangsu and Sichuan provinces) in June and November, respectively, and recorded snail abundances and mortalities. There was a strong compensatory feedback of density on reproduction for both marshland and mountainous-area snails (Figure 3). Pooling all data, the combined evidence for density feedback models was > 99% for *O. hupensis*, based on the sum of the *w*AIC*_c _*for the Ricker-logistic and Gompertz-logistic models, with most support for the Gompertz (Table 1).

**Table 1:** Contrasting four demographic density feedback models for *O.hupensis *snail density

Setting	Model	AIC*_c_*	ΔAIC*_c_*	*w*AIC*_c_*	%DE
Overall	RW	252.568	72.111	2.1939E-16	0.0
	EX	234.944	54.487	1.4731E-12	30.2
	RL	197.414	16.957	0.0002	66.1
	GL	180.457	0.000	**0.9998**	**75.1**
Jiangsu	RW	122.090	46.979	5.8346E-11	0.0
	EX	97.919	22.808	1.0344E-05	61.2
	RL	80.208	5.097	0.0725	81.2
	GL	75.111	0.000	**0.9275**	**84.3**
Sichuan	RW	131.071	42.189	6.8969E-10	0.0
	EX	129.474	40.593	1.5323E-09	13.6
	RL	106.567	17.685	0.0001	66.3
	GL	88.882	0.000	**0.9999**	**82.5**

RW, random walk; EX, exponential growth; RL, Ricker-logistic; GL, Gompertz-logistic; %DE, % deviance explained (goodness of fit). Highest-ranked models are shown in boldface

Model strength of evidence based on Akaike's information criterion corrected for small samples (AIC*_c_*).

The slope of *r *versus log (*N_t_*) (Gompertz-logistic) relationship for the pooled *O. hupensis *population was -0.796 (standard error [SE] = 0.084) (equation 10). The slopes of the relationship were -0.502 (SE = 0.082) and -1.133 (SE = 0.115) for the Jiangsu and Sichuan subspecies, respectively (equations 11 and 12), suggesting a stronger compensatory feedback mechanism in the Sichuan subspecies (Figure 3).(10)(11)(12)

The effective population growth rates of mountainous snails in low-density groups are about 3-4 times higher than those observed in the marshland.

**Figure 4:**
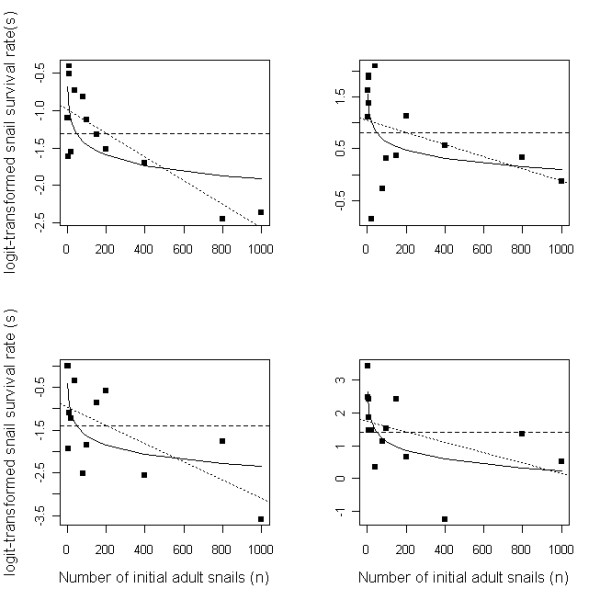
**Three contrasting models of *O. hupensis *survival rate (*s*) as a function of density: density-independent (DI - dashed line), linear decline (DDL - dotted line) and log-linear decline (DDLL - solid line)**. See also Table 2. Upper panels: Jiangsu population (left: first generation; right: second generation); lower panels: Sichuan population (left: first generation; right: second generation).

The survival probability of the snails in higher-density groups was lower than that in lower-density groups, and there was strong evidence for either a Ricker-like (linear) or Gompertz-like (log-linear) decline in logit-transformed survival rate for both generations and both study populations (Table 2 and Figure 4). However, the first generation demonstrated more linear decline, while the second generation had a more log-linear decline in survival with density (Figure 4). These results suggest that adult survival is one of the principal component vital rates driving the demographic feedback patterns.

**Table 2:** Contrasting three models of *O.hupensis *snail survival rate as a function of density: density-independent (DI), linear decline (DDL) and log-linear decline (DDLL)

Setting	Generation	Model	AIC*_c_*	ΔAIC*_c_*	*w*AIC*_c_*	%DE
Jiangsu	G1	DI	30.148	12.297	0.0022	0.0
		DDL	17.851	0.000	**0.9792**	67.2
		DDLL	25.763	7.912	0.0187	42.3
	G2	DI	41.111	1.165	0.2866	0.0
		DDL	41.828	1.883	0.2002	16.9
		DDLL	39.946	0.000	**0.5132**	27.4
Sichuan	G1	DI	45.009	4.368	0.0682	0.0
		DDL	40.641	0.000	**0.6053**	42.2
		DDLL	41.876	1.235	0.3265	36.9
	G2	DI	47.623	5.387	0.0602	0.0
		DDL	48.007	5.771	0.0497	18.9
		DDLL	42.236	0.000	**0.8901**	46.3

DDL, linear density effect; DDLL, log-linear density effect; DI, density-independent; %DE, % deviance explained (goodness of fit). Highest-ranked models are shown in boldface.

Mode1 strength of evidence based on Akaike's information criterion corrected for small samples (AIC*_c_*).

## Discussion

First experiences with implementing a new integrated control strategy for schistosomiasis control in P.R. China are promising [8,9,11]. However, we speculate that control efforts could be further enhanced by rigorously implementing intermediate host-snail suppression until snail densities are below a pre-set detection threshold. Our models revealed a strong feedback mechanism, whereby extremely low-density habitats following control can in fact produce the largest subsequent pulses of snails, thus potentially continuing or even exacerbating schistosomiasis outbreaks [39]. Hence, snail control, in concert with the new comprehensive schistosomiasis control strategy [7], would also act to suppress natural fluctuations of the disease in humans and animals, especially in areas of low prevalence. There is a need to continue suppression given that in lake districts, fishermen and river-transportation workers regularly come into contact with contaminated freshwater bodies (schistosome eggs and, most importantly, schistosome cercariae, which are the infective stages), and thus perpetuate local outbreaks. In mountainous regions, buffalo (*Bubalus bubalis*) act as the major definitive host of *S. japonicum*, and are still used as the core farming animal in agriculture, and hence cannot be replaced immediately by more modern mechanical farming equipment. It follows that the persistence of suitable snail habitats, and hence intermediate host snails, calls for sustained control via chemical mollusciciding and environmental management.

A better understanding of the population dynamics of intermediate host snails holds promise to render snail control more effective. With regard to the work presented here, the following conclusions can be drawn. First, we showed that effective mollusciciding must reduce snail density to less than two specimens per 0.5 m^2 ^because even at these low densities, populations can increase quickly to over 50 times initial abundance given low initial competition among individuals. Second, the only molluscicide currently recommended by the World Health Organization is niclosamide [40]. At present in P.R. China, the most commonly used formula of molluscicide is 50% wettable powder of niclosamide ethanolamine salt (Bayluscide) [41]. A recent systematic review showed that snail mortality is around 88% using a 50% wettable powder of niclosamide ethanolamine salt post spraying for 15 days [42]. There is considerable concern that, if mollusciciding is only attempted once by spraying niclosamide in the field, the few surviving snails will engender a rapid re-bound in the months to come. We therefore recommend applying molluscicide at least twice yearly and, if financial and technical resources allow, even more frequently.

Although the population growth dynamic of snails in both marshland and mountainous regions followed the same pattern, we observed some notable differences. For example, the slope of the regression model for *O. hupensis *in the mountainous areas is considerably greater than that for the marshland (-1.133 versus -0.502), which translates to the prediction that mountainous snails are likely more sensitive to variation in density than marshland strains at low population sizes. As a result, *O. hupensis *from mountainous regions seem more elusive to control than marshland snails because of the high rebound rate expected subsequent to mollusciciding. Compared to marshland *O. hupensis*, our findings suggest that snail control in mountainous regions must be implemented with utmost vigilance to reduce population rate of increase that tends constantly towards exponential growth at low densities. If possible, environmental management in mountainous regions should be recommended with the ultimate goal to eliminate suitable snail habitats. Recent work indicated that schistosomiasis is re-emerging in mountainous regions, perhaps explained by the overall high rate of snail rebound [39].

Although we could not test the component contribution of reproduction explicitly given the confounding effects of a semelparous, annual-breeding species (i.e. a generation lasts only until the next breeding cycle), it is clear that adult survival contributes a large component to the demographic response. We also found that the decline in survival with density was greatest and linear for the first generation, but weaker and log-linear for the second (Figure 4). This might arise from differing environmental conditions experienced by each generation, or differential mortality patterns, or a combination of both. Thus, examining patterns of density feedback across a wider range of experimental conditions will further enhance our understanding of snail rebound potential following control.

Of course, snail population size is not solely a product of density feedback. In addition to intrinsic influences, the distribution, extent and density of *O. hupensis *depend on a complex interaction of different environmental factors, of which temperature, rainfall and flooding are the three most important [43]. Future modelling must consider these factors and the potential influence of environmental changes (e.g. climate change) on long-term predictions of the cost-effectiveness of control interventions.

## Competing interests

The authors declare that they have no competing interests.

## Authors' contributions

GJY conceived the study and wrote the first version of the manuscript. XNZ, LPS, FW, BZ and DCQ helped in the field experiments in Jiangsu and Sichuan provinces. CJAB contributed to data analysis. XNZ, JU and CJAB revised the manuscript. All of authors read, contributed to, and approved the final version of the manuscript.
